# Structural characterization of a novel cyclic 2,3-diphosphoglycerate synthetase involved in extremolyte production in the archaeon *Methanothermus fervidus*

**DOI:** 10.3389/fmicb.2023.1267570

**Published:** 2023-11-16

**Authors:** Simone A. De Rose, Michail N. Isupov, Harley L. Worthy, Christina Stracke, Nicholas J. Harmer, Bettina Siebers, Jennifer A. Littlechild, The HotSolute consortium, Bettina Siebers, Christopher Bräsen, Christina Stracke, Benjamin Meyer, Michail N. Isupov, Nicholas J. Harmer, Simone Antonio De Rose, Jennifer Ann Littlechild, Elizaveta Bonch-Osmolovskaya, Sergey Gavrilov, Ilya Kublanov, Daniela Monti, Erica Ferrandi, Eleonora Dore, Felix Müller, Jacky Snoep

**Affiliations:** ^1^Henry Wellcome Building for Biocatalysis, Biosciences, Faculty of Health and Life Sciences, University of Exeter, Exeter, United Kingdom; ^2^Biosciences, Faculty of Health and Life Sciences, University of Exeter, Exeter, United Kingdom; ^3^Department of Molecular Enzyme Technology and Biochemistry, Environmental Microbiology and Biotechnology, and Centre for Water and Environmental Research, University of Duisburg-Essen, Essen, Germany; ^4^Living Systems Institute, Faculty of Health and Life Sciences, University of Exeter, Exeter, United Kingdom

**Keywords:** extremolyte, cyclic 2, 3-diphosphoglycerate, X-ray structure, thermophiles, synthetase

## Abstract

The enzyme cyclic di-phosphoglycerate synthetase that is involved in the production of the osmolyte cyclic 2,3-diphosphoglycerate has been studied both biochemically and structurally. Cyclic 2,3-diphosphoglycerate is found exclusively in the hyperthermophilic archaeal methanogens, such as *Methanothermus fervidus*, *Methanopyrus kandleri*, and *Methanothermobacter thermoautotrophicus*. Its presence increases the thermostability of archaeal proteins and protects the DNA against oxidative damage caused by hydroxyl radicals. The cyclic 2,3-diphosphoglycerate synthetase enzyme has been crystallized and its structure solved to 1.7 Å resolution by experimental phasing. It has also been crystallized in complex with its substrate 2,3 diphosphoglycerate and the co-factor ADP and this structure has been solved to 2.2 Å resolution. The enzyme structure has two domains, the core domain shares some structural similarity with other NTP-dependent enzymes. A significant proportion of the structure, including a 127 amino acid N-terminal domain, has no structural similarity to other known enzyme structures. The structure of the complex shows a large conformational change that occurs in the enzyme during catalytic turnover. The reaction involves the transfer of the γ-phosphate group from ATP to the substrate 2,3 -diphosphoglycerate and the subsequent S_N_2 attack to form a phosphoanhydride. This results in the production of the unusual extremolyte cyclic 2,3 -diphosphoglycerate which has important industrial applications.

## Introduction

Osmolytes are small organic molecules that accumulate within cells as a response to conditions of stress. These molecules increase the thermodynamic stability of cellular proteins and nucleic acids without compromising their native functional activities (Yancey et al., 1982). Osmolytes are tolerated within cells at concentrations, from millimolar to 1–2 molar, depending on the extracellular osmolarity (Brown, 1976).

Extremolytes are osmolytes from extremophilic organisms that are adapted to environmental extremes including high pressure, extremes of pH, high salinity, and high or low temperatures (Raddadi et al., 2015). Extremolytes have many biotechnological and industrial applications, including their use as additives for storage of high value macromolecules enzymes, drugs and antibodies (Barth et al., 2000; Cruz et al., 2006; Lentzen and Schwarz, 2006). They are also important as food and cosmetic product ingredients (Buenger and Driller, 2004; Graf et al., 2008; Marini et al., 2014).

In recent years there has been increased interest in the biotechnological application of extremolytes, most prominently ectoine and hydroxyectoine, due to their well established production and purification methods (Becker and Wittmann, 2020). Ectoines have excellent protein function preserving properties, which has led to their recognition as chemical chaperones. This has fostered the development of an industrial scale biotechnological production process for their exploitation in skin care and medicinal products (Czech et al., 2018).

**SCHEME 1:**
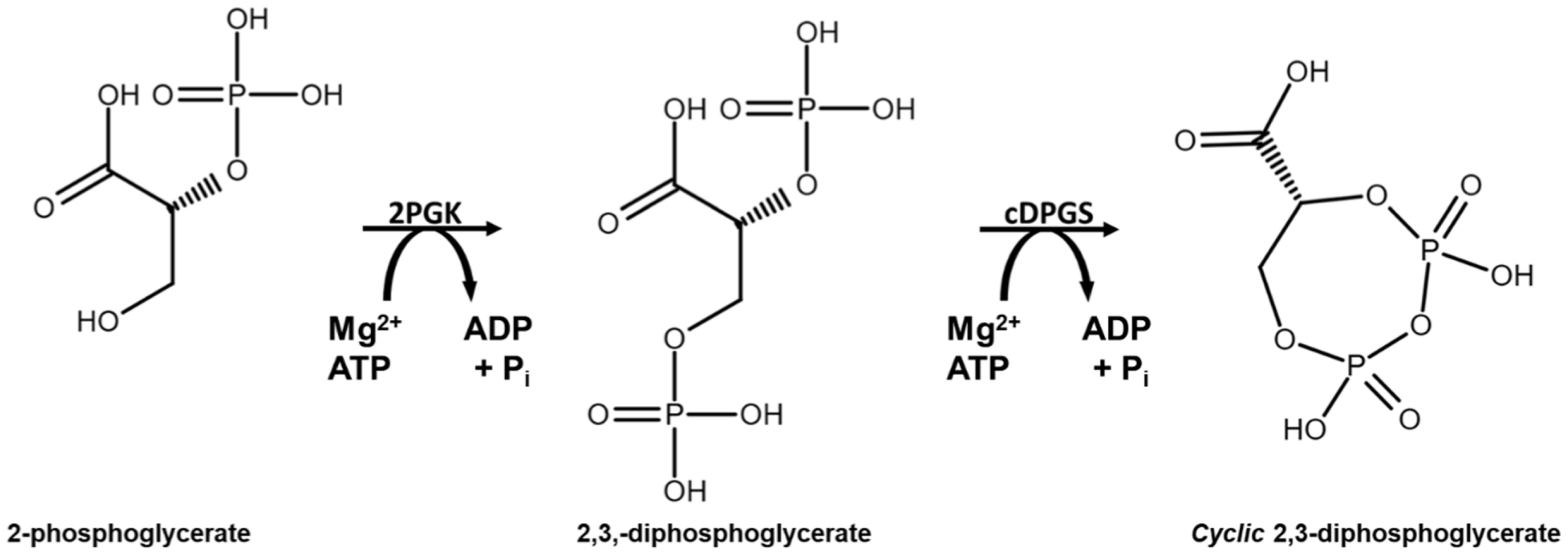
Reaction scheme for production of cyclic 2,3-diphosphoglycerate using the enzymes 2PGK and cDPGS derived from the archaeon *M. fervidus*.

Another extremolyte is cyclic 2,3-diphosphoglycerate (cDPG). cDPG has been exclusively found in the hyperthermophilic archaeal methanogens such as *Methanothermus fervidus, Methanopyrus kandleri* and *Methanothermobacter thermoautotrophicus*, at concentrations 0.3–1.1 M (Hensel and König, 1988; Ciulla et al., 1994; Matussek et al., 1998). cDPG is synthesized by a two-step enzymatic pathway from the glycolytic intermediate 2-phosphoglycerate (2PG). The process requires two enzymes, 2-phosphoglycerate kinase (2PGK) which forms 2,3-di-phosphoglycerate (2,3DPG) from 2PG, and cyclic di-phosphoglycerate synthetase (cDPGS) which cyclizes 2,3DPG to form the extremolyte cDPG (Lehmacher et al., 1990; Scheme 1).

In the native archaeal methanogenic species, cDPG biosynthesis is triggered by an increase in the growth temperature (Lehmacher et al., 1990). The accumulation of this extremolyte in the cells is correlated with the optimum growth temperature of the archaeal species. The concentration of cDPG increases from 70 mM in *M. thermoautotrophicum* (65°C), to 300 mM in *M. fervidus* (84°C), and 1 M in *M. kandleri* (98°C) (Shima et al., 1998). An additional role has been suggested for intracellular cDPG as a phosphate and energy storage compound (Sastry et al., 1992; Van Alebeek et al., 1994; Lentzen and Schwarz, 2006). Since the cDPGS reaction is exergonic at cellular concentrations, cDPG accumulation is favored thermodynamically until this reaction reaches equilibrium (Shima et al., 1998). cDPG appears to play a role in the thermoprotection of proteins, and increased thermostability has been demonstrated for several model enzymes in its presence. In addition, cDPG protects plasmid DNA against oxidative damage by hydroxyl radicals (Lentzen and Schwarz, 2006). It can also function as a superoxide scavenger, with efficiency reaching one third of that of the antioxidant ascorbic acid (Valentão et al., 2002). Both 2PGK and cDPGS are activated by potassium ions. High concentrations (0.3–0.5 M) of these ions have been reported to increase the activity of 2PGK and cDPGS by 2.4 and 1.4 fold, respectively, (Lehmacher et al., 1990; Van Alebeek et al., 1991, 1994). In physiological conditions, the activation is more modest and has been reported to be around 10% for both enzymes. Interestingly, NaCl at concentrations up to 1 M does not affect enzyme activities (Lehmacher et al., 1990).

In a recent study, we have established a process to produce cDPG using the thermophilic bacterium *Thermus thermophilus* as a whole-cell factory (De Rose et al., 2021). A protein BLAST (Altschul et al., 1990) search of the cDPGS and 2PGK sequences against the Protein Data Bank (PDB) revealed that there are no other known protein structures which share significant sequence similarity, making these enzymes of novelty and interest. Here, we report the high-resolution X-ray structures of cDPGS in its apoform and in complex with its substrate 2,3 DPG and its cofactor ADP. The overall structure is unique and only part of one domain has been shown to structurally align with the structures of other known unrelated enzyme activities. The details of the cDPGS structure described in this paper provides some important insight into its reaction mechanism.

## Results

### Expression and purification

The gene encoding the cDPGS was successfully cloned in the pLATE51 expression vector in frame with the N-terminal His6x-tag sequence and under the control of the lactose inducible promoter. The His-tagged cDPGS protein was successfully over-expressed in a soluble form in *Escherichia coli* BL21 (DE3). cDPGS was purified from the cell extracts by Ni^2+^-NTA affinity chromatography with a recovery yield of 20 mg L^−1^. This was followed by size exclusion chromatography (SEC), that showed that the purified cDPGS elutes in a dimeric form of ~100 kDa (Supplementary Figure S1). As previously reported (Lehmacher et al., 1990; Van Alebeek et al., 1991, 1994) the presence of at least 300 mM KCl is essential for the purification of cDPGS to maintain the enzyme correctly folded and in an active state. Protein purification using 500 mM NaCl instead of KCl lead to poor recovery.

The purified cDPGS was assayed for its thermal stability using differential scanning fluorimetry (DSF) Vivoli et al., 2014). However due to the high thermostability of the enzyme its melting only starts to appear at 95°C. Due to the instrument limitations it was not possible to obtain a precise apparent T_m_ value for this enzyme. This demonstrates that cDPGS is a highly thermostable enzyme with a melting temperature above 95°C (Supplementary Figure S2).

### Activity assay

**SCHEME 2:**
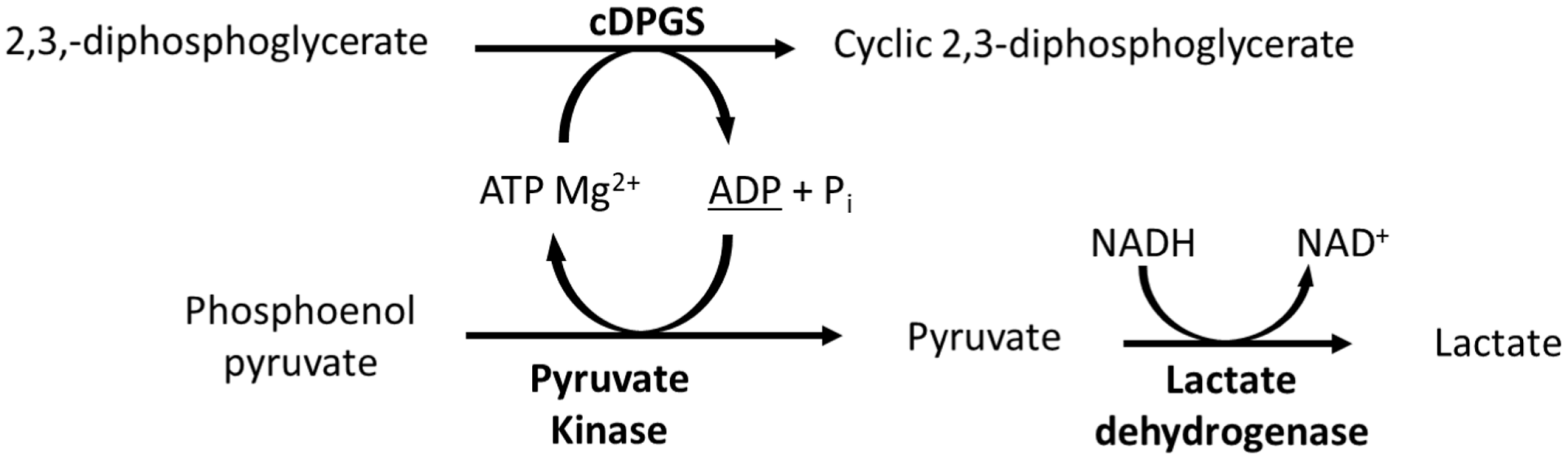
Schematic representation of the linked assay used to indirectly monitor the activity of cDPGS. The ADP production was determined spectrophotometrically by monitoring of the oxidation of NADH to NAD^+^ at 340 nm.

**Figure 1:**
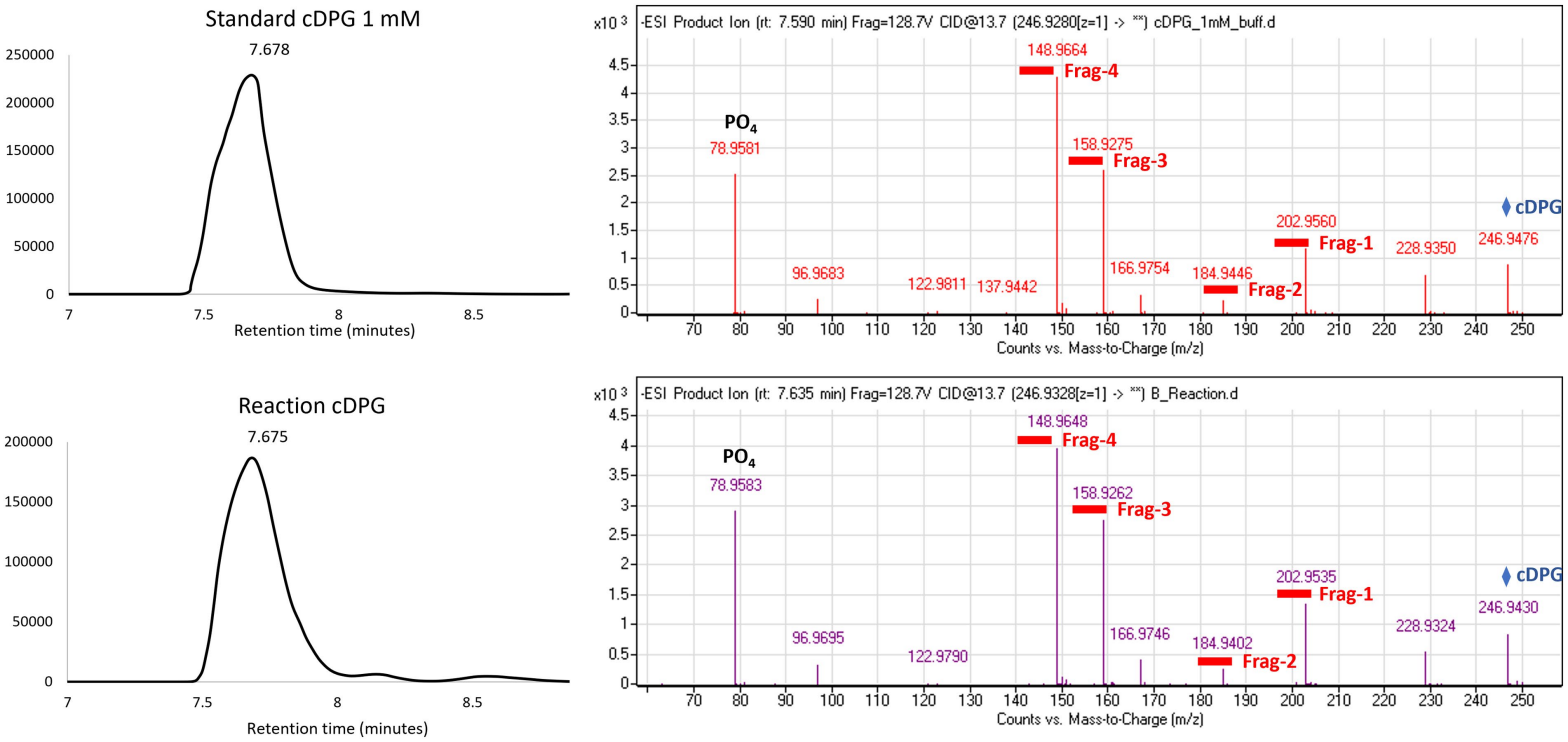
Extracted ion chromatogram and MS (Mass Spectrometry) fragmentation patterns for the control (top panel) and the cDPG reaction (bottom panel). The MS fragmentation confirms the identity of the molecule, with an accuracy of 27.2089 ppm and 20.7295 ppm, respectively, for the standard and reaction. The whole molecule mass (cDPG: 246.9 Da) is indicated by the blue diamond, while the four fragments’ masses are underlined in red.

To confirm the expression of an active correctly folded protein suitable for crystallization studies, the activity of the cDPGS was measured by monitoring the ADP formation using a linked assay with pyruvate kinase (PK) and l-lactate dehydrogenase (LDH) (Scheme 2). The cDPGS was found to be active with a specific activity of 0.039 U/mg (Supplementary Figure S3). The direct production of cDPG was monitored by HPLC–MS to confirm the correct identification of the product (Figure 1 and Supplementary Figure S4).

### Crystal structure and overall topology of apo-cDPGS

The cDPGS crystallized readily in many screening conditions and the structure of the apo-cDPGS was solved by the single wavelength anomalous dispersion (SAD) method using data collected from a selenomethionine protein crystal. The space group was determined to be *I*222 with a single protomer of cDPGS in the asymmetric unit. The structure was refined to 1.7 Å resolution and was found to contain the entire polypeptide chain. This has been refined to *R* and *R*_free_ values of 17.7 and 21.3%, respectively (Table 1).

**Table 1:** cDPGS data collection and refinement statistics.

cDPGS	Ligand free	ADP 2,3 DPG complex
Data collection statistics		
Beamline	IO3 Diamond	IO3 Diamond
Wavelength (Å)	0.9763	0.9763
Space group	I222	P1
Unit Cell Parameters a, b, c (Å)	74.5, 105.8, 157.0	71.1, 71.3, 103.3
a, β, g (°)	90.0, 90.0, 90.0	96.9, 103.4, 99.1
Resolution range (Å)^a^	42.71–1.64	69.62–2.23
Total reflections^a^	822,404	167,272
Unique reflections^a^	362,595 (15356)	92,153 (4558)
Completeness (%)^a^	100.0 (99.9)	97.7 (97.0)
Multiplicity^a^	5.0	1.8
R_meas_ (%)^a^^,^^b^	0.110	0.115
/^a^	8.0 (0.3)	6.3 (0.2)
CC_1/2_^a^^,^^c^	0.99 (0.28)	0.98 (0.38)
Wilson B-factor^d^ (Å^2^)	37.8	70.3
Refinement statistics		
R_work_	0.177	0.216
R_free_	0.213	0.256
No. of protomers in a.u.	1	4
Number of atoms	4,232	14,537
Macromolecules	3,817	14,416
Ligands/Metal ions	119	77
Solvent	293	50
Number of protein residues	460	460
RMS bond lengths (Å)	0.010	0.005
RMS bond angles (°)	1.58	1.36
Ramachandran favored (%)^e^	98.03	97.81
Ramachandran outliers (%)^e^	0.0	0.0
Clashscore^e^	4.04	0.21
Average B-factor protein (Å^2^)	37.942	74.855
Average B-factor ligands (Å^2^)	47.274	96.300
Average B-factor solvent (Å^2^)	45.201	51.956
RCBS PDB code	8ORK	8ORU

a Values for the highest resolution shell are given in parentheses.

b Rmeas = Σh [m/(m—1)]1/2 Σi|Ih,i— < Ih > |/Σh ΣiIh,i.

c CC1/2 is defined in Karplus and Diederichs (2012).

d Wilson B-factor was estimated by SFCHECK (Vaguine et al., 1999).

e The Ramachandran statistics and clashscore statistics were calculated using MOLPROBITY (Chen et al., 2010).

**Figure 2:**
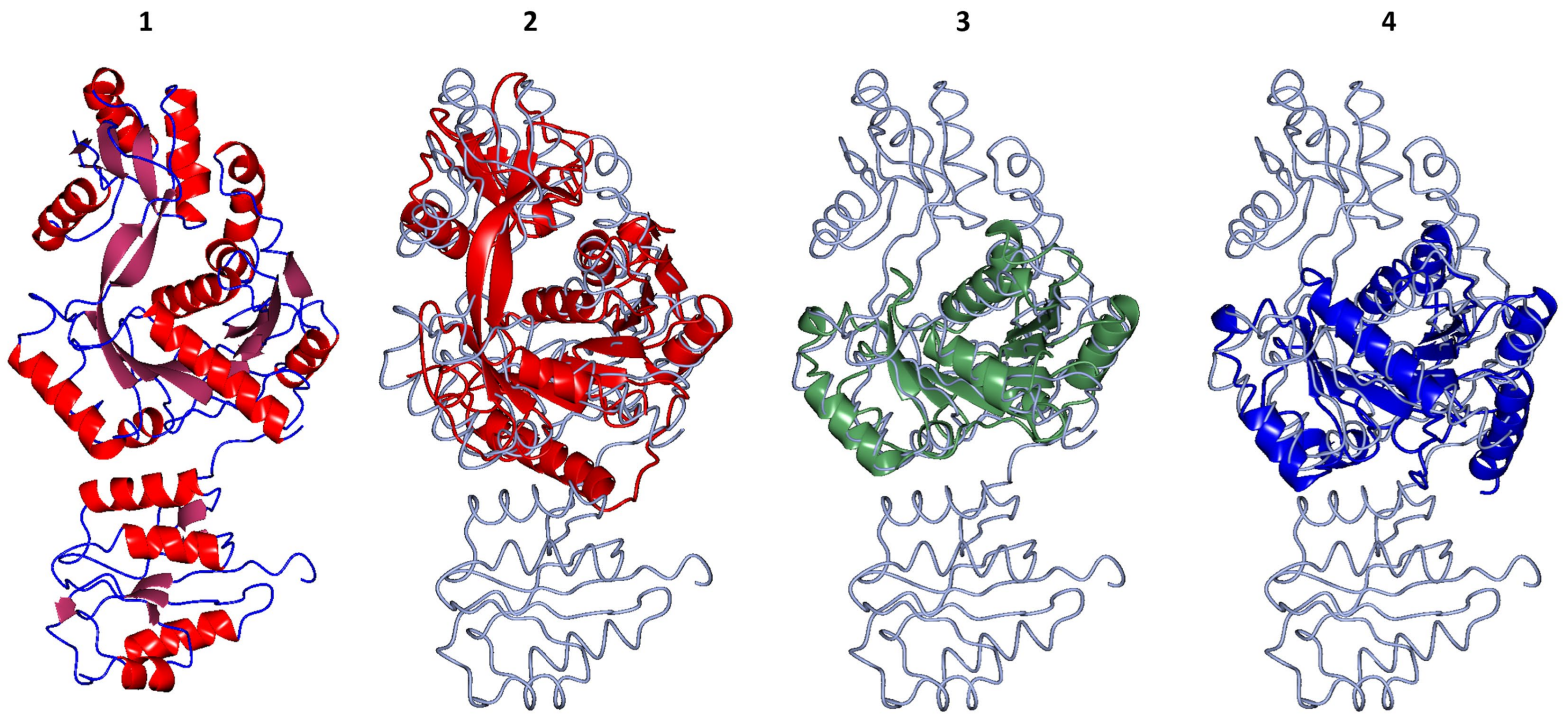
Structural superposition of the cDPGS monomer with its three closest structural homologs as reported by the DALI server (Holm and Laakso, 2016; Holm, 2020). From left to right: **(1)** cDPGS monomer colored by secondary structure elements, α-helices (red) β-sheets (pink), loops and turns (blue), in accordance with the topology diagram in Supplementary Figure S5, **(2)** *Aquifex aeolicus* tetraacyldisaccharide 4′-kinase (red), **(3)** *Klebsiella pneumoniae* urease accessory protein UreG (green), and **(4)** *Helicobacter pylori* hydrogenase/urease nickel incorporation protein H (blue). In panels 2, 3 and 4 cDPGS is depicted as a thin cyan tube. Figure prepared with CCP4mg (McNicholas et al., 2011).

The overall structure of the cDPGS showed only limited similarity to other known protein structures according to the DALI structural comparison server (Holm and Laakso, 2016; Holm, 2020). The results showed a few structural homologs with a DALI Z score of >7.0. The best scoring homologs were an *Aquifex aeolicus* tetraacyldisaccharide 4′-kinase (PDB entry 4EHX, Z score = 15.9), a *Klebsiella pneumoniae* urease accessory protein UreG (PDB entry 5XKT, Z score = 14.0) and the *Helicobacter pylori* hydrogenase/urease nickel incorporation protein H (PDB entry 4LPS, Z score = 13.2). Structural alignment with these proteins showed a core region that is conserved amongst other NTP-dependent enzymes (Figure 2). However, a portion of the structure, including a 127 amino acid N-terminal domain, shows no structural similarity to other known structures in the PDB. A protein BLAST (Altschul et al., 1997) search of the N-terminal domain sequence against the PDB did not produce any hits. The DALI server did not find any known protein domains with a fold close to that of the N-terminal domain of cDPGS.

The structure of cDPGS can be split into two domains. The smaller N-terminal domain contains a six-stranded β-sheet of mixed type with direction + + + + + − and connectivity -1x,-1x,3x,1x,1 (Richardson, 1981) flanked by 4 α-helices (α1–α4). The β-sheets of the two N-terminal domains in the cDPGS dimer form a large intersubunit 12 strand β-sheet on the molecular dyad. The C-terminal domain of cDPGS roughly aligns with the unusual P-loop kinase tetraacyldisaccharide 4′-kinase (LpxK) from *A. aeolicus* (Emptage et al., 2012). The cDPGS C-terminal domain and LpxK show the same Rossmann like α/β/α sandwich fold connected by two twisted, antiparallel β-strands. The larger C-terminal domain (residues 136–460) contains a twelve-stranded β-sheet (β7 – β18) surrounded by twelve α-helices. The strand direction is − − − − + + + + + + + − and connectivity is 2x,2,-1,-2x,-2x,-1x,-1x,-3,-1x,2x,1x (Supplementary Figure S5).

**Figure 3:**
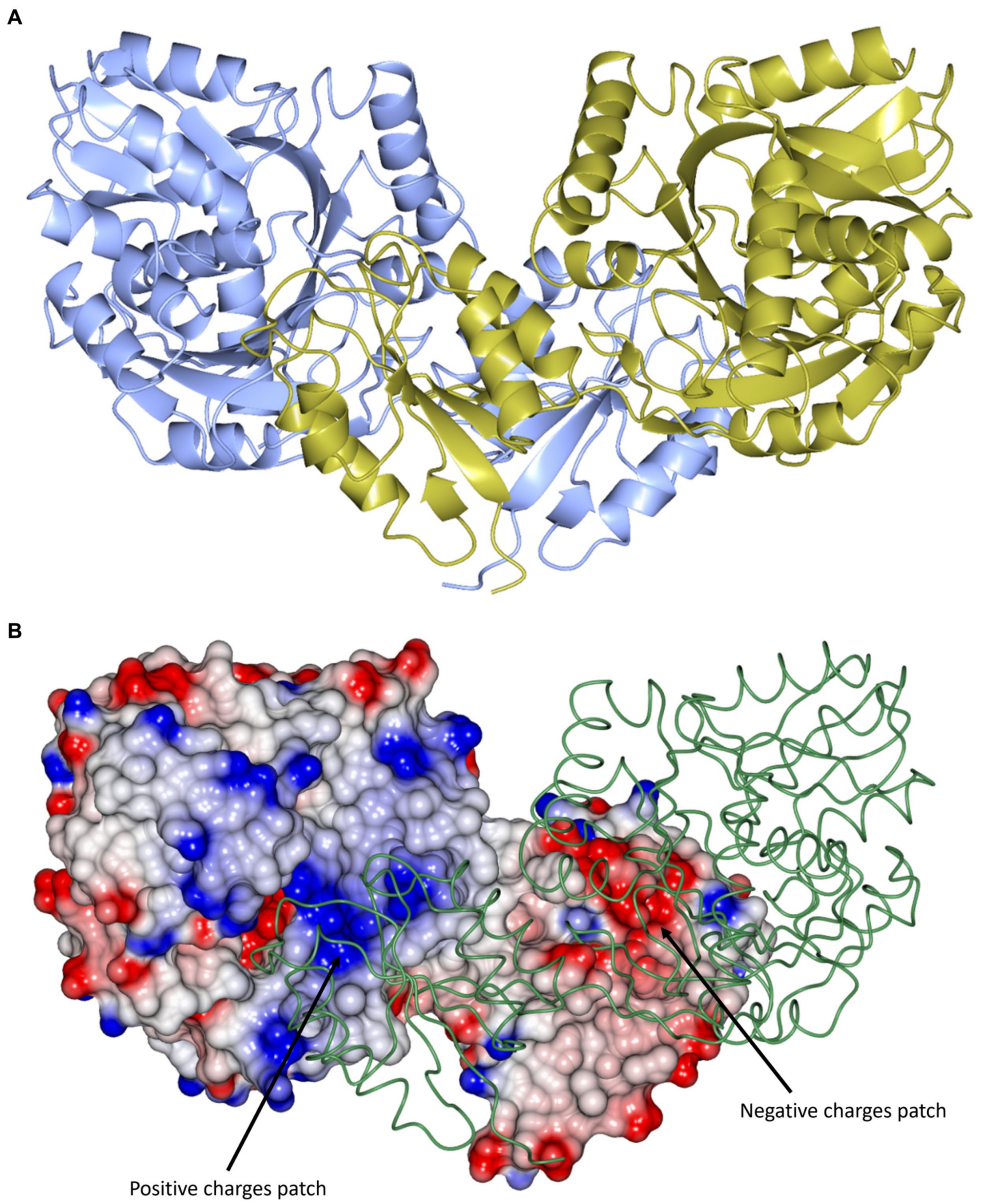
**(A)** A cartoon representation of the apo-cDPGS dimer viewed perpendicular to a molecular dyad with the two protomers in cyan and gold. **(B)** A cartoon diagram showing the hydrophobic interactions at the dimer interface of cDPGS. For clarity one protomers is shown in space filling mode with electrostatic surface potential and the other as a thin green tube. The areas of positive charge are shown in blue, with the areas of negative charge in red and the hydrophobic surfaces are represented in white, the two major charges patches involved in the dimer interaction are highlighted by black arrows. Figure prepared with CCP4mg (McNicholas et al., 2011).

The cDPGS structure shows a tight dimer that buries a surface area of 2844.1 Å^2^, accounting for 13.7% of the total solvent accessible area of the protomer (Figure 3A). The interface is stabilized by 36 hydrogen bonds and 24 salt bridges between the interacting subunits as estimated by PISA (Krissinel and Henrick, 2007). These include an intersubunit 12-strand β-sheet formed by the N-terminal domains. The dimer interface of cDPGS clearly shows a large positively charged patch at the interface of the C-terminal domain that interacts with the negatively charged N-terminal domain of the opposing molecule (Figure 3B). The high thermal stability of cDPGS appears to be due to both hydrophobic interactions and a high number of hydrogen bonds and ion pairs. Similar interactions have previously been observed in other thermostable proteins with temperature optima up to approximately 75°C (Sayer et al., 2012; Ferrandi et al., 2018).

**Figure 4:**
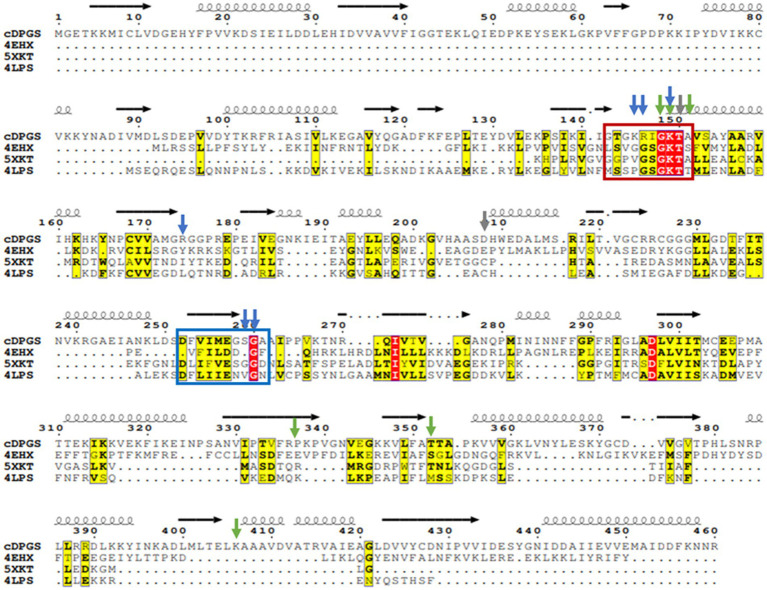
Multiple sequence alignment of cDPGS with its three closest structural homologs. Red and yellow residues have a high degree of conservation among the orthologues where “red” indicates absolute conservation, “yellow” indicates conservation between similar groups. The cDPGS secondary structure is depicted on top of the sequence as spring and arrows along with the location of the Walker A/P-loop and Walker B motifs in the red and blue boxes, respectively. The accession numbers of the cDPGS orthologues in order are as follows: 4EHX *Aquifex aeolicus* tetraacyldisaccharide 4′-kinase; 5XKT *Klebsiella pneumoniae* urease accessory protein UreG; 4LPS *Helicobacter pylori* hydrogenase/urease nickel incorporation protein H. Arrowheads indicate the residue directly interacting with the ADP (blue), magnesium (gray) and 2,3 DPG (green). The alignment was generated using ClustalO (Sievers et al., 2011), and visualized with Esprit3 (https://espript.ibcp.fr) (Robert and Gouet, 2014).

Multiple sequence alignments using the results from the structural comparison server revealed a number of highly conserved residues (Figure 4) that tend to cluster around the nucleotide and ligand binding sites, particularly a P-loop/ Walker A motif GxxGxGK[T/S] (Walker et al., 1982). This loop typically binds the phosphate groups of phosphorylated ribonucleotides and catalyzes phosphoryl transfer (Romero Romero et al., 2018).

### Crystal structure of ADP/Mg^2+^ bound cDPGS

To elucidate the substrate binding residues of cDPGS, the structure of the 2,3 DPG, ADP/Mg^2+^ bound enzyme complex was solved by molecular replacement to a resolution of 2.2 Å resolution, resulting in *R*_work_ and *R*_free_ values of 21.5 and 25.6%, respectively (Table 1).

**Figure 5:**
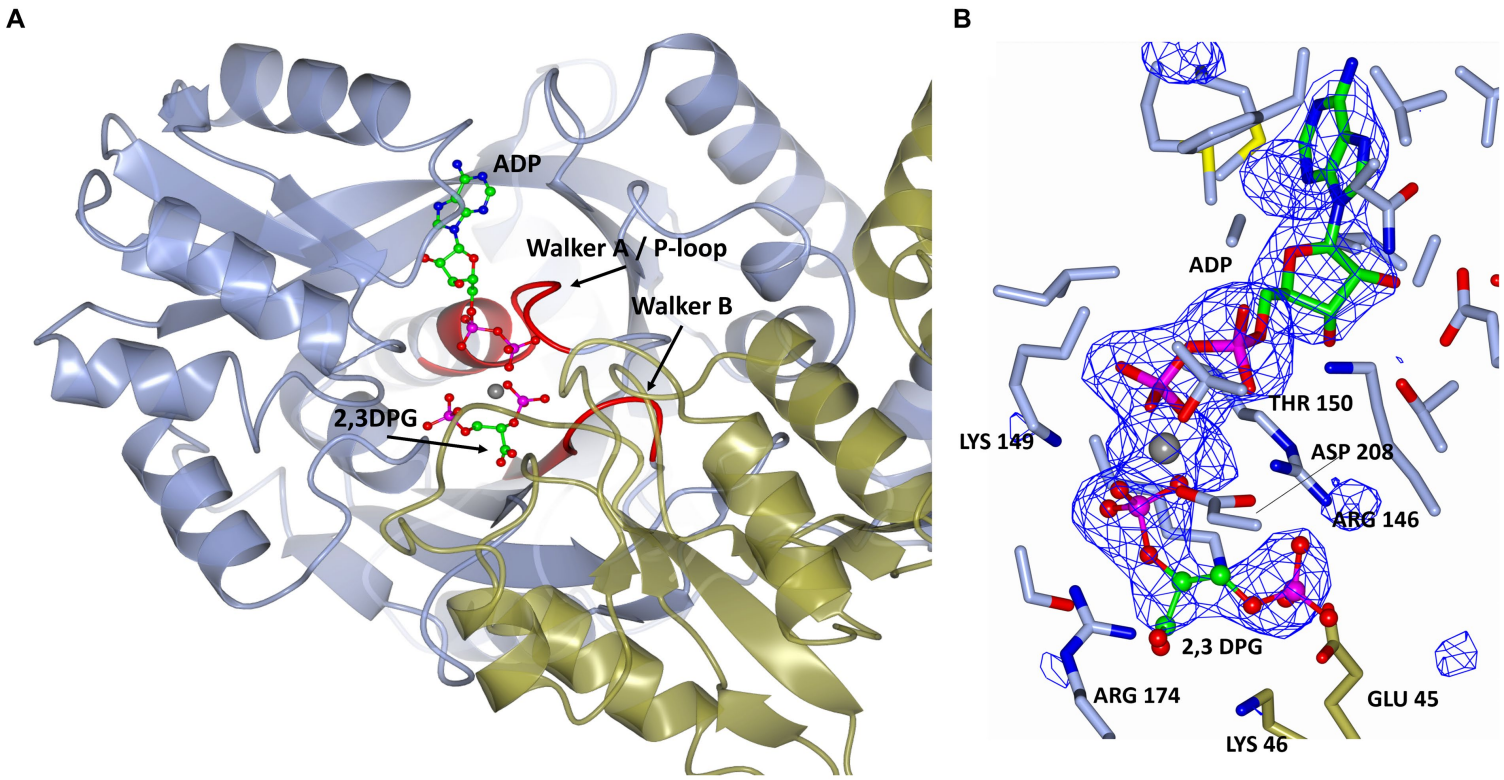
**(A)** A cartoon representation of the ADP and 2,3 DPG ligands bound to cDPGS. The two subunits are shown in cyan and green. ADP and 2,3 DPG are shown as a ball-and-stick model (carbon, green; oxygen, red; nitrogen, blue; phosphorus, pink). The Mg^2+^ atom is depicted as a gray sphere. The Walker A/ P-loop and Walker B loop are shown in red. **(B)** A view of 2,3 DPG and ADP bound in the active site of cDPGS showing neighboring protein residues. The magnesium atom is shown as a gray sphere; ADP and 2,3 DPG are shown as a ball-and-stick models (carbon, green; oxygen, red; nitrogen, blue; phosphorus, pink). Residues belonging to molecule A of the dimer are shown in ice blue while Glu45 and Lys46 from molecule B are shown in green. The Fo-Fc difference map calculated with ADP, Mg^2+^ and 2,3 DPG at zero occupancy is shown in blue contoured at 3.0σ. This clearly shows the presence of the ligand 2,3 DPG. Figure prepared with CCP4mg (McNicholas et al., 2011).

The 2,3 DPG binding site is located between the C-terminal domain of the one protomer and the N-terminal domain of the other protomer of the dimer (Figure 5A). The presence of the bound ligands is confirmed by the Fo-Fc difference map calculated with ADP Mg^2+^ and 2,3 DPG at zero occupancy. The ADP and Mg^2+^ are clearly resolved and reside in the pocket formed within the C-terminal domain (Figure 5B). The 2,3 DPG is clearly resolved in only three of the four protomers making up the asymmetric unit of the crystal. A phosphate molecule has been modeled in the active site of the 4th protomer. The Walker A/P-loop surrounds the pyrophosphate moiety of the ADP while coordinating with the Mg^2+^ atom. The adenosine moiety is bound in a pocket formed by α5, α14, β13, β14 and β15. The Walker B loop borders the 2,3 DPG binding site without directly interacting with it.

**Figure 6:**
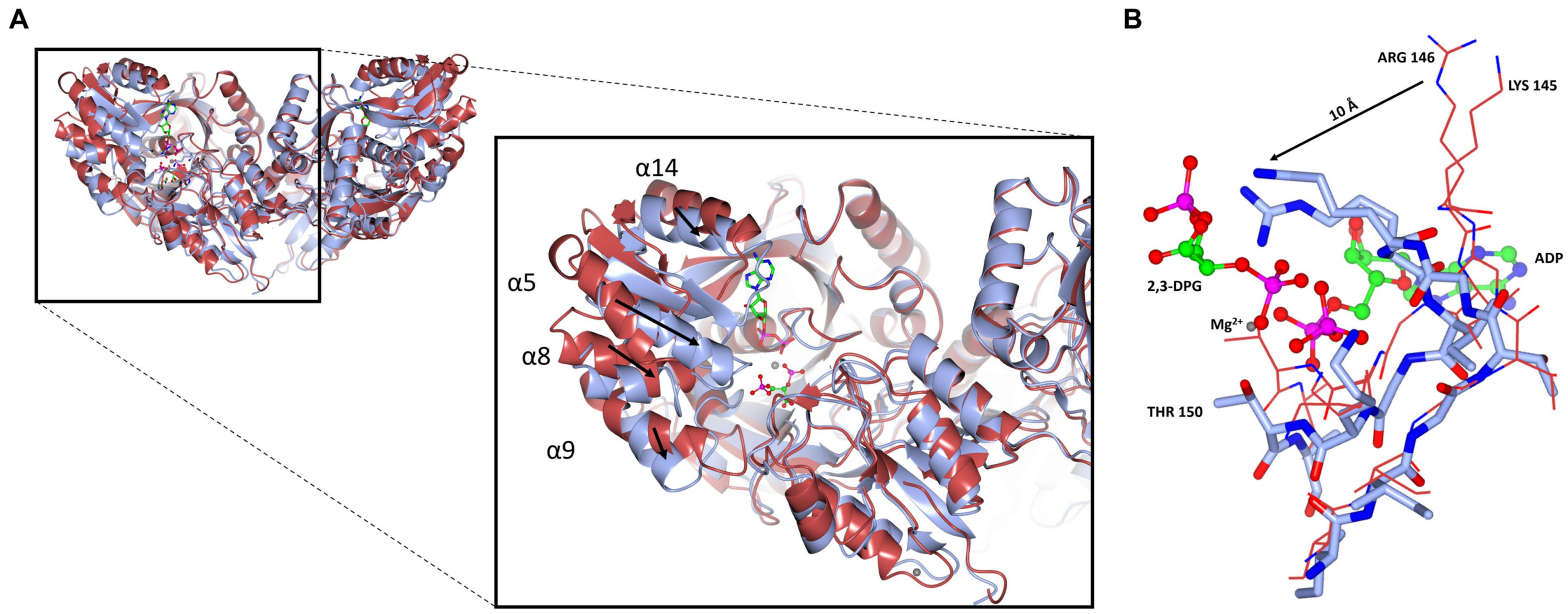
Cartoon representation of the domain closure induced by binding of ADP/Mg^2+^. **(A)** A super-imposition of the apo-cDPGS (red) with the ADP and 2, 3DPG bound to cDPGS (cyan). Black arrows indicate the direction of movement induced by the conformational changes. **(B)** Close-up of the same super-imposition showing the local movements (black arrow) that follow ADP and 2,3DPG binding. Figure prepared with CCP4mg (McNicholas et al., 2011).

**Figure 7:**
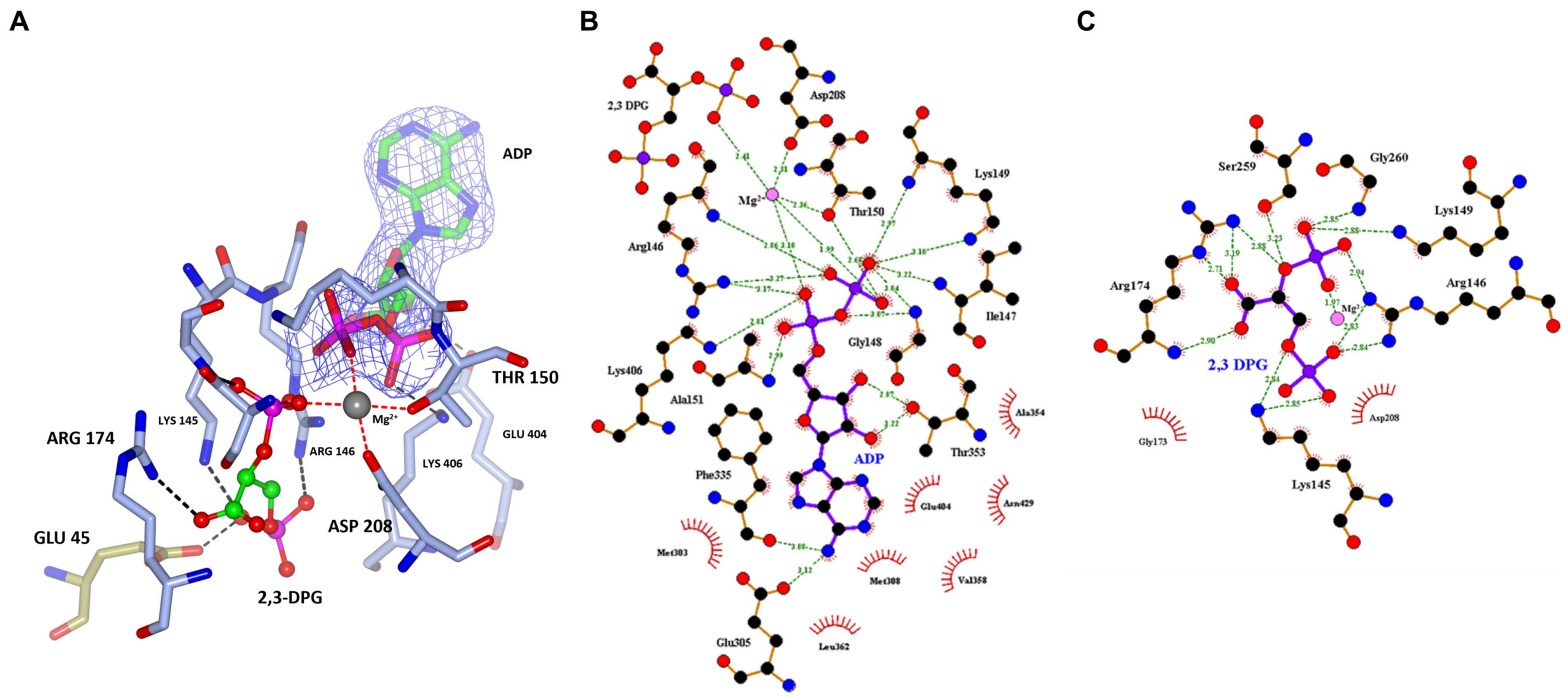
**(A)** A view of 2,3 DPG and ADP bound in the active site of cDPGS showing residues involved in the stabilization of the substrates. The magnesium atom is shown as a gray sphere; ADP and 2,3 DPG are shown as a ball-and-stick model (carbon, green; oxygen, red; nitrogen, blue; phosphorus, pink); the coordination bonds to the Mg^2+^ are shown as red dotted lines while hydrogen bonds are shown as black dotted lines. Residues belonging to molecule A of the dimer are shown in ice blue while GLU45 from molecule B is shown in green, the 2Fo-Fc electron density of the ADP molecule contoured at 1.5σ is shown in blue for better visual separation from the 2,3 DPG molecule and surrounding residues. Schematic overview of the **(B)** nucleotide/magnesium binding site and **(C)** 2,3 DPG pocket. Hydrogen bonds are indicated with green dashes and their distances indicated. Panel **(A)** prepared with CCP4mg (McNicholas et al., 2011), panels **(B,C)** generated using LigPlot^+^ (Wallace et al., 1995; Laskowski and Swindells, 2011).

A superposition of the apo- and ADP/Mg^2+^ bound forms of cDPGS reveals a large movement of several α-helices that close around the nucleotide, which highlight the significant conformational changes that accompany ligand binding (Figure 6A). The helices α5, α8, α9 and α14 show a motion of up to 8.5, 7.0, 6.7, and 2.6 Å, respectively. The loops formed by residues 186–192, 396–400 and 420–427 make up the “hinge” regions. A closer look at the active site revealed that the closure is triggered by the binding of the substrate and ADP which brings the Walker A/P-loop close enough to contribute to the stabilization of 2,3 DPG. Residues K145 and R146 move closer to the substrate to form multiple hydrogen bonds with it (Figure 6B). The 2,3DPG is also stabilized by E45 from the opposing subunit (Figures 7A,B). The pyrophosphate moiety of ADP is held in place by an intricate network of hydrogen bonds with backbone amide nitrogen atoms, various side chains, and water molecules (Figure 7C). The magnesium atom is octahedrally coordinated by the side chain oxygens of D208, T150, the O3B oxygen of ADP, O9 oxygen of 2,3DPG and two water molecules. The domain movement causes the interaction of several residues with the ADP. Outside the P-loop, residues E305, P335, T353 and E404 appear to have a significant role in ATP binding since they form hydrogen bonds to the adenosine, ribose hydroxyl groups, or the α-phosphate of ADP.

**SCHEME 3:**
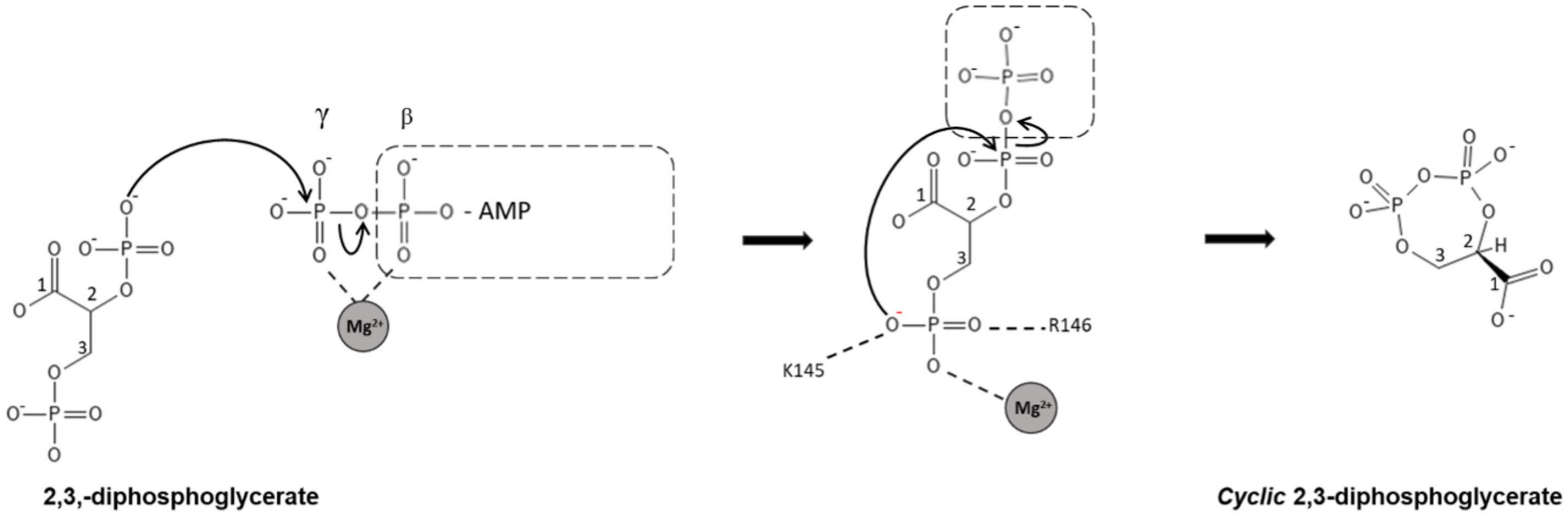
Schematic representation of the proposed mechanism for the synthesis of cDPG. In step 1, the cDPG 2-phosphate attacks the γ-phosphate of ATP, forming the 2-pyrophosphate intermediate. In the second step, the 3-phosphate attacks the pyrophosphate, releasing inorganic phosphate and forming cDPG. Both reactions are S_N_2 reactions with simultaneous formation and breaking of the P-O bonds. The highly negatively charged transition states are stabilized by magnesium and positively charged amino acid side chains.

Based on the apo structure and the ADP/Mg^2+^ complex structures, we propose a classical phosphoryl transfer mechanism for cDPGS as observed in other NTP dependent enzymes (Matte et al., 1998; Guixé and Merino, 2009; Gerlits et al., 2015). The conformational changes that accompany binding of ATP and 2,3 DPG bring the ATP into the proximity of 2,3 DPG in a sterically and energetically strained conformation, forming a ‘near attack complex’ (Bruice, 2002). Positively charged residues (K145, R146, K149, K406) and the magnesium ion will shield the negative charges on the phosphates, allowing the reactants to approach. The domain closure creates a hydrophobic environment allowing the phosphoryl transfer from the ATP γ-phosphate to the 2-phosphate of the 2,3 DPG through an S_N_2 reaction, with the transition state stabilized by the magnesium ion, to form the intermediate. A second S_N_2 reaction is initiated by attack of the 3-phosphate of the intermediate on the 2-pyrophosphate, releasing an inorganic phosphate. This step is facilitated by the magnesium ion, K145, and R146 providing positive charges to stabilize the transition state. Release of phosphate makes the reaction highly exergonic under standard conditions, providing the energy for the formation of the cDPG phosphoanhydride (Scheme 3).

## Discussion

cDPGS has been long known for its role in the biosynthesis of cDPG. This extremolyte which is only found naturally in specific methanogenic archaea such as *M. fervidus* which was first isolated in 1981 by Karl Stetter (Stetter et al., 1981). This organism has attracted interest since it has the smallest genome known to date for a free-living archaeon, coding for only 1,311 proteins and 50 RNA genes (Martínez-Cano et al., 2015). *M. fervidus* only grows under strict anaerobic conditions and obtains its energy by the reduction of carbon dioxide with hydrogen to produce methane. It is known to use cDPG up to a 0.3 M intracellular concentration for protein stabilization at temperatures of 75°C and above. Since its discovery cDPG has been the subject of a limited number of research publications (Hensel and König, 1988; Lehmacher et al., 1990; Van Alebeek et al., 1991; Lehmacher and Hensel, 1994; Van Alebeek et al., 1994; Matussek et al., 1998) which have focused mainly on its identification, biochemical characterization and its production pathway.

This paper describes the cloning and over-expression in *E. coli* of cDPGS, involved in the cDPG production. This enzyme has been characterized both biochemically and structurally. The unique structure of cDPGS is made up of an N-terminal domain which is crucial for the stabilization of the active dimeric structure and the ligand interactions, and a C-terminal domain that contains a P-loop kinase fold as part of its structure. From a comparison with the closest structural homologs highlighted in the multiple sequence alignment shown in Figure 4 little can be observed apart from a few conserved regions that match with the P-loop kinases fold. The crystal structures of both the apo- and ADP/Mg^2+^-bound forms of cDPGS have allowed us to understand its substrate specificity and mechanism, and to provide an insight into the features that determine its extreme thermostability. The functional dimeric form of cDPGS is seen to undergo a conformational change upon binding of ADP/Mg^2+^ and 2,3DPG during the cyclisation reaction. The super-imposition of the apo-cDPGS and its ADP/Mg^2+^ bound complex structures reveal a closure of the C-terminal domain around the ADP product in the active site of the enzyme.

We propose that cDPGS remains in the ‘open’ conformation until the substrate and ATP are bound in the correct orientation for turnover to occur, which then triggers domain closure providing a hydrophobic environment for the cyclisation reaction. The enzyme then is required to return to its ‘open’ conformation to release the product and ADP, as described for other ATP dependent enzymes (Davies et al., 1993; Auerbach et al., 1997; Bowler, 2013; Murillo-López et al., 2019; Recabarren et al., 2019).

More information could be derived from the crystal structure of the cDPGS in complex with cDPG, as well as the crystal structure of the ATP transition state that could be mimicked using a transition state analog such as AlF_3_ where fluorine substitutes the ATP γ-phosphate (Baxter et al., 2006; Cliff et al., 2010). However, it has not been possible to date to crystallize these complexes.

This unusual ‘primitive’ cDPGS enzyme which is found only in methanogenic hyperthermophilic archaea has naturally evolved to form the unique protein structure described in this paper. This allows the production of cDPG, a natural extremolyte, to stabilize the proteins and DNA for growth and survival of the organism in extreme environments. The cDPG, due to its known protective roles for proteins (Hensel and Jakob, 1993; Borges et al., 2002) and DNA (Lentzen and Schwarz, 2006) has industrial applications for a variety of high value cosmetic and healthcare products. The biosynthesis of cDPG has already been demonstrated in a cascade reaction using the two enzymes 2PGK and cDPG both *in vitro* or *in vivo* (De Rose et al., 2021). The optimisation of these methodologies will allow the scale-up of the production of this extremolyte for new biotechnological applications.

## Materials and methods

### Cloning, expression, and purification

The genes coding for *Mf*-cDPGS in its *E. coli* codon optimized version was kindly donated by the Siebers lab (Essen Germany) and sub-cloned into the pLATE51 vector (ThermoFisher) in frame with a N-terminal 6x His-Tag. pLATE51/cDPGS (Supplementary Figure S7) was introduced into *E. coli* DH5α (New England Biolabs, C2987H) using standard techniques. The plasmid was purified and sequenced to confirm the presence of the gene. The plasmid pLATE51/cDPGS (Addgene: 201561) was introduced into *E. coli* BL21 competent cells (Agilent Technologies, 230,132) according to the manufacturer’s instructions.

The transformants obtained were grown in LB supplemented with 100 μg/mL ampicillin (LB_amp100_) medium (50 mL) overnight and then inoculated in 0.5 L LB _amp100_ at 37°C and 200 rpm. When the OD_600_ reached 0.5–0.6, gene expression was induced by the addition of 0.5 mM IPTG, and the culture was maintained at 25°C for 24 h. Cells were harvested by centrifugation (4,750 *g* for 30 min), and frozen at −20°C before proceeding with the protein purification.

A selenomethionine derivative (SeMet) of the enzyme was also produced in *E. coli* using the protocol described by Studier. Briefly the protein was expressed in PASM-5052 auto induction media, supplemented with 100 μg/mL ampicillin, 200 μg/mL each of 17 aa (no C, Y, M), 10 μg/mL methionine, 125 μg/mL selenomethionine and 100 nM vitamin B_12_ (Studier, 2005).

The frozen cell paste expressing cDPGS was thawed and re-suspended in 50 mM Tris–HCl pH 7.5, 300 mM KCl, and 20 mM imidazole. The cells were disrupted by sonication at 10 μm (Soniprep150; MSE) on ice for 4 min and the cell debris was removed by centrifugation at 24000 *g* at 4°C for 30 min. The clarified cell lysate was then heat-treated at 60°C for 30 min before being centrifuged at 24000 *g* at 4°C for 30 min to remove any denatured proteins. The protein was purified using a 1 mL HisTrap FF crude column (Cytiva) using a two-step gradient. Step one increased the imidazole concentration to 40 mM for 10 column volumes (CV), whilst step two increased the concentration to 300 mM imidazole for 20 CV. The eluate from the second step was applied to a calibrated Superdex 200 HiLoad 16/60 gel filtration column (Cytiva) and eluted with one column volume of 20 mM Tris–HCl pH 7.5, 300 mM KCl, at 1.0 mL min^−1^.

### Enzyme characterization

#### Indirect activity assay by coupled reaction

The enzymatic activity was determined using a coupled assay (Scheme 2). This couples the ADP formation from ATP to NADH oxidation via pyruvate kinase (PK) (Merck, 10109045001) and L-lactate dehydrogenase (LDH) (Merck, 427217). The assay mixture (200 μL) contained 50 mM MES/KOH pH 6.5 with 2.5 mM MgCl_2_, 400 mM KCl, 2.5 mM ATP, 0.5 mM NADH, 3.7 μg purified cDPGS, 8 U PK, 4 U LDH, and 2 mM phosphoenolpyruvate. The reactions were started by the addition of 2,3DPG and the oxidation of NADH was followed in an Infinite 200 pro spectrometer (Tecan) at 340 nm. All measurements were performed in triplicates at 37°C since higher temperatures could destabilize the coupling enzymes PK and LDH.

#### Differential scanning fluorimetry

Differential scanning fluorimetry DSF was used as a method to monitor protein unfolding with increasing temperature. In this method, proteins are incubated with a fluorescent dye which alters its fluorescence upon binding to the hydrophobic regions of the proteins. The protein dye mixture is then heated, and the fluorescence monitored as the heat rises. The unfolding of the protein and exposure of hydrophobic parts of the protein gives rise to a characteristic pattern of the fluorescence as a function of temperature (Vivoli et al., 2014). The DSF samples were prepared at the following concentrations in a final volume of 20 μL: 1 mg/mL protein, 10 mM HEPES pH 7.0, 150 mM NaCl, 8 × SYPRO Orange dye (Invitrogen). All samples were prepared in triplicate. The fluorescence was measured using a StepOne quantitative PCR machine (Applied Biosystems, Foster City, CA, USA) while heating the samples in a gradient from 25 to 99°C over 40 min. Measurements were taken every 0.37°C. The DSF curves obtained were used to calculate the midpoint temperature of the unfolding transition (T_m_) using the differential of each DSF curve calculated using the Protein Thermal Shift software package (Applied Biosystems).

#### Direct activity assay by HPLC-MS

The direct activity of cDPGS was determined from the detection of cDPG formed from 2,3 DPG. 0.1 mg/mL of enzyme solution was incubated with 100 μL of a substrate mixture containing 25 mM Tris–HCl pH 8.0, 50 mM MgCl_2_, 50 mM ATP, 250 mM 2,3-DPG and 300 mM KCl at 65°C for 60 min. The reaction was stopped with the addition of a 2:1 ratio of ice-cold acetonitrile. Samples were incubated for 10 min on ice and centrifuged at 13000 *g* for 10 min to precipitate all of the enzyme before the supernatant was collected and transferred into a HPLC vial. Samples were kept at 4°C and analyzed by HPLC–MS within 24 h. Reactions were performed in triplicate. The control reaction was performed in the same condition without the enzyme.

### Analytical methods

#### LC-QTOF-MS polar metabolite profiling

cDPG profiling was performed using a Q-TOF 6520 mass spectrometer (Agilent Technologies) coupled to a 1,200 series Rapid Resolution HPLC system. 5 μL of sample extract was loaded onto an Agilent Infinity Lab Poroshell 120 HILIC-Z 2.7 μm, 2.1 × 250 mm analytical column. For detection using negative ion mode, mobile phase A comprised 100% water with 10 mM ammonium acetate and 5 μM medronic acid and mobile phase B was 90% acetonitrile with 10 mM ammonium acetate and 5 μM medronic acid. All solvents were LC-MS grade. The following gradient was used: 0 min – 95% B; 5 min – 65% B; 10 min – 50% B; 11 min – 95% B; 15 min – 95% B followed by 1 min post time. The flow rate was 0.25 mL min^−1^ and the column temperature was held at 25°C for the duration of the measurement. The source conditions for electrospray ionization were as follows: gas temperature was 325°C with a drying gas flow rate of 9 L min^−1^ and a nebulizer pressure of 35 psg. The voltages for the capillary, fragmentor, and skimmer were 3.5 kV, 115 V, and 70 V, respectively. Scanning was performed using the auto MS/MS function at 5 scans sec^−1^ for precursor ion surveying and 4 scans sec^−1^ for MS/MS, with a sloped collision energy of 3.5 V/100 Da with an offset of 5 V. MassHunter qualitative analysis (version B.07.00) was used to identify potentially interesting compounds with similar masses to the sugar phosphate of interest.

#### Crystallization and structure solution

A sample of cDPGS from the right side of Peak 2 _Supplementary Figure S1 corresponding to the dimeric form was concentrated to ~10 mg/mL using a 10 kDa membrane Vivaspin (Vivascience) and microbatch and sitting drop crystallization trials were set up using an Oryx8 crystallization robot (Douglas Instruments) using the Morpheus^™^ (Molecular Dimensions) protein crystallization screens. Microbatch trials were set in hydrophobic plates (VB-SILVER-2, Douglas Instruments), with a final drop volume of 0.5 μL. The droplet contained a 50:50 ratio of protein solution to screen and was covered with Al’s oil (50,50 mix of silicon and paraffin oils) before being stored at 20°C. For sitting drop trials, hydrophilic plates were used (MRC96T-PS, SwissCL) with a drop volume of 1 μL the droplets contained 40:60 and 60:40 protein to screen ratio for each of the conditions.

cDPGS native crystals appeared within 1 week in most of the conditions of the Morpheus screen. The crystals were harvested straight from the crystallization droplet and plunged into liquid nitrogen. Preliminary data were collected to 7.0 Å resolution at 100 K on the Diamond beamline IO3. Further crystals grown with selenomethionine enriched protein diffracted to a higher resolution of 1.7 Å in space group *I*222 on the Diamond beamline IO3. The data were processed using DIALS (Waterman et al., 2016) within the Xia2 pipeline (Winter et al., 2013). These data were used for solution of the structure with single anomalous diffraction (SAD) method using CRANK-2 (Skubák and Pannu, 2013) in CCP4CLOUD (Krissinel et al., 2022).

The selenomethionine enriched crystals grew in condition F7 of the Morpheus screen consisting of 0.12 M Monosaccharides mix, 0.1 M Buffer System 2 pH 7.5 and 30% v/v Precipitant Mix 3. The substrate bound crystals grew (Supplementary Figure S6) in condition E12 of the Morpheus screen consisting of 0.12 M Ethylene glycols mix, 0.1 M Buffer System 3 pH 8.5 and 37.5% v/v of Precipitant Mix 4, with 10 mM 2,3 DPG, 20 mM MgCl_2_ and 20 mM ADP.^1^

The substrate bound crystals diffracted to 2.2 Å in space group P1. Data were processed and scaled using XDS (Kabsch, 2010) and AIMLESS (Evans and Murshudov, 2013) in the Xia2 pipeline (Winter et al., 2013). The structure was solved by molecular replacement using MOLREP (Vagin and Teplyakov, 2010) using the native structure coordinates. All further data and model manipulations were carried out using the CCP4 suite of programs (Winn et al., 2011; Agirre et al., 2023). The resulting structure was subjected to refinement in REFMAC5 (Murshudov et al., 2011) and rebuilding in COOT (Emsley et al., 2010; Table 1). The PISA software (Krissinel and Henrick, 2007) was used for oligomeric state analysis of the cDPGS models.

## Data availability statement

The datasets presented in this study can be found in online repositories. The names of the repository/repositories and accession number(s) can be found at: https://www.rcsb.org/, PDB 80RK, PDB 80RU.

## Author contributions

SR: Methodology, Writing – original draft, Writing – review & editing, Formal analysis, Investigation. MI: Investigation, Methodology, Writing – original draft, Writing – review & editing, Data curation, Software, Supervision, Validation. HW: Data curation, Investigation, Writing – review & editing. CS: Writing – review & editing, Methodology, Resources. NH: Investigation, Validation, Writing – review & editing. BS: Writing – review & editing, Methodology, Conceptualization, Project administration, Funding acquisition. JL: Writing – review & editing, Conceptualization, Funding acquisition, Methodology, Project administration, Resources, Supervision, Writing – original draft.

## HotSolute

List of the HotSolute consortium participants: Bettina Siebers, bettina.siebers@uni-due.de; Christopher Bräsen, christopher.braesen@uni-due.de; Christina Stracke, christina.stracke@uni-due.de; Benjamin Meyer, benjamin.meyer@uni-due.de; Michail N. Isupov, misupov@exeter.ac.uk; Nicholas J. Harmer, N.J.Harmer@exeter.ac.uk; Simone Antonio De Rose, S.A.De-Rose@exeter.ac.uk; Jennifer Ann Littlechild, J.A.Littlechild@exeter.ac.uk; Elizaveta Bonch-Osmolovskaya, elizaveta.bo@gmail.com; Sergey Gavrilov, sngavrilov@gmail.com; Ilya Kublanov, kublanov.ilya@gmail.com; Daniela Monti, daniela.monti@scitec.cnr.it; Erica Ferrandi, erica.ferrandi@scitec.cnr.it; Eleonora Dore, eleonora.dore@scitec.cnr.it; Felix Müller, felix.mueller@evonik.com; and Jacky Snoep, jacky.snoep@mac.com.

## Footnotes

^1^ https://calibrescientific.com/en/products/MDL-MD1-47
