# Supplementary material for: Structural characterization of a novel cyclic 2,3-diphosphoglycerate synthetase involved in extremolyte production in the archaeon Methanothermus fervidus
Source: Front Microbiol. 2023 Nov 16;14:1267570. doi: 10.3389/fmicb.2023.1267570 (PMC10690619; doi:10.3389/fmicb.2023.1267570)
Supplement: Supplementary file 1 [file Data_Sheet_1.pdf]

**Supplementary materials**

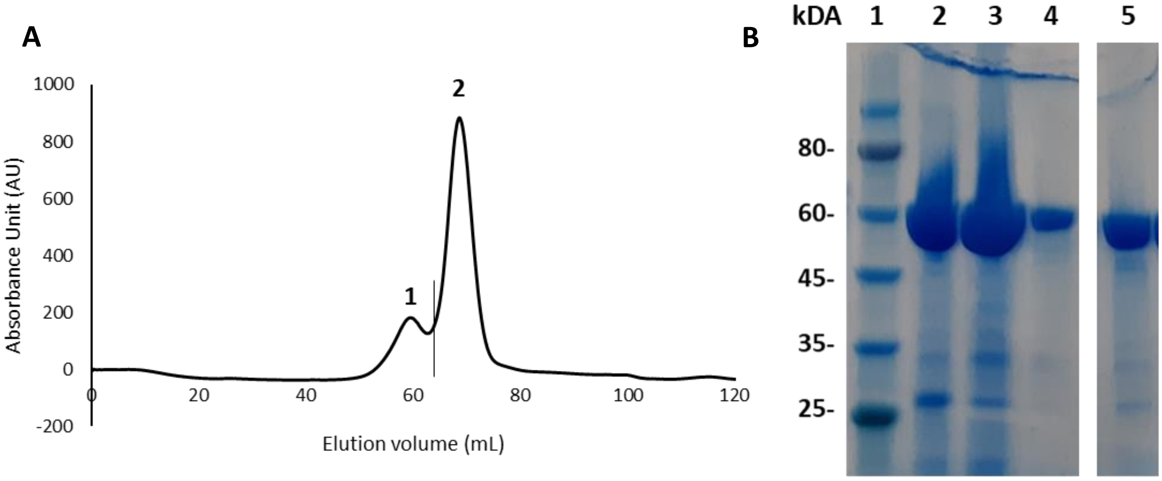


Figure S1: A) Size exclusion chromatography elution profile (Superdex 200 HiLoad 16/600) of cDPGS. A first peak 1 eluting at 60 mL corresponds to cDPGS with an oligomeric state for a suggested tetramer, while the second peak 2 eluting at 70 mL is cDPGS with an apparent molecular mass corresponding to a dimeric state of the protein (∼100 kDa). A sample from peak 2 was used for the crystallization studies. B) SDS-PAGE showing the purification of cDPGS. Lane 1: Molecular weight markers, lane 2 and 3: Ni-NTA elution, lane 4: SEC Peak 1 and lane 5: SEC Peak 2.

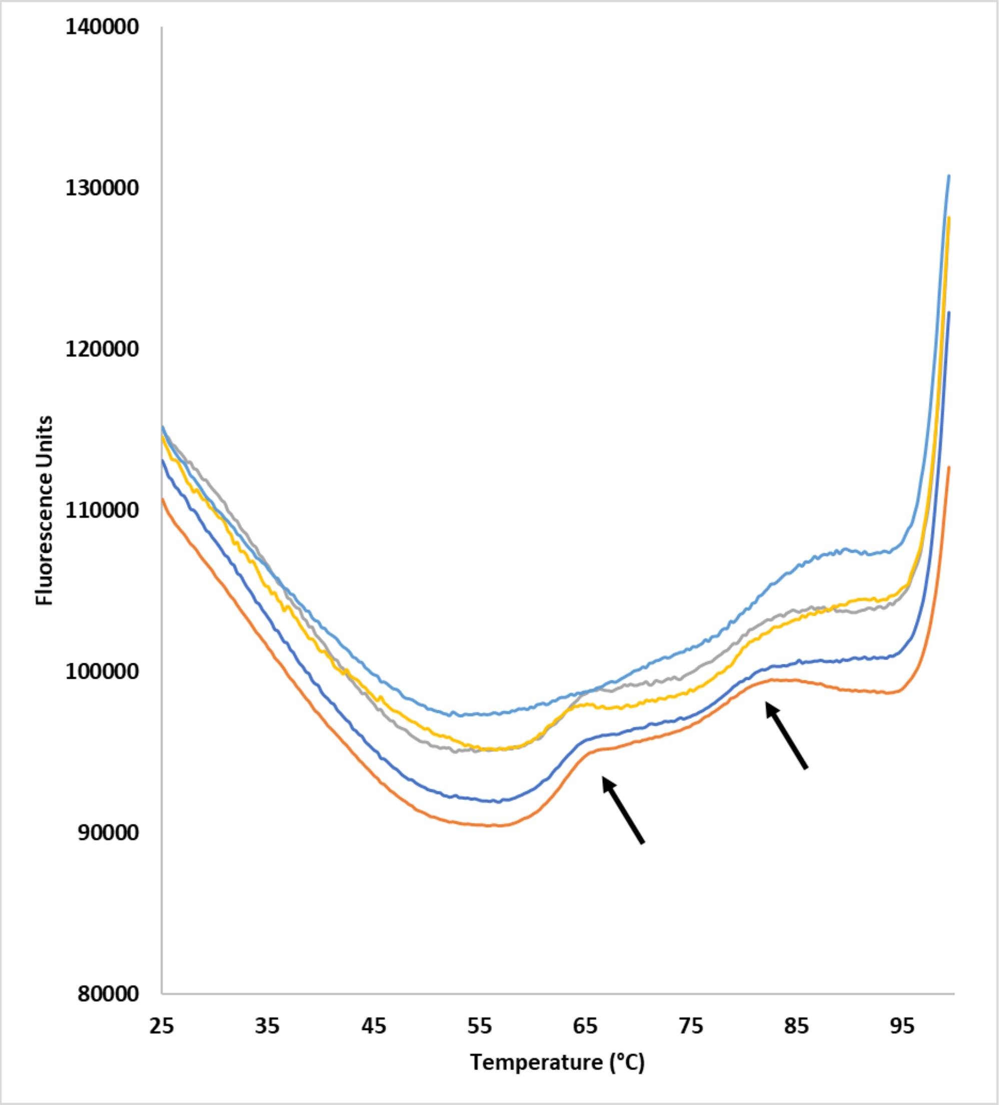


Figure S2: Results of differential scanning fluorimetry of cDPGS. Five replicate traces are shown. The fluorophore used (SYPRO Orange) is temperature sensitive and so the fluorescence initially drops with temperature. Minor fold relaxation events occur as indicated by the arrows around 70 °C and 85 °C. These suggest a conformational change of the protein structure to facilitate activity as often observed for thermophilic enzymes. The overall protein unfolding event is initiating between 95 °C and 99 °C (the temperature limit of the instrument) but has clearly not reached a plateau.

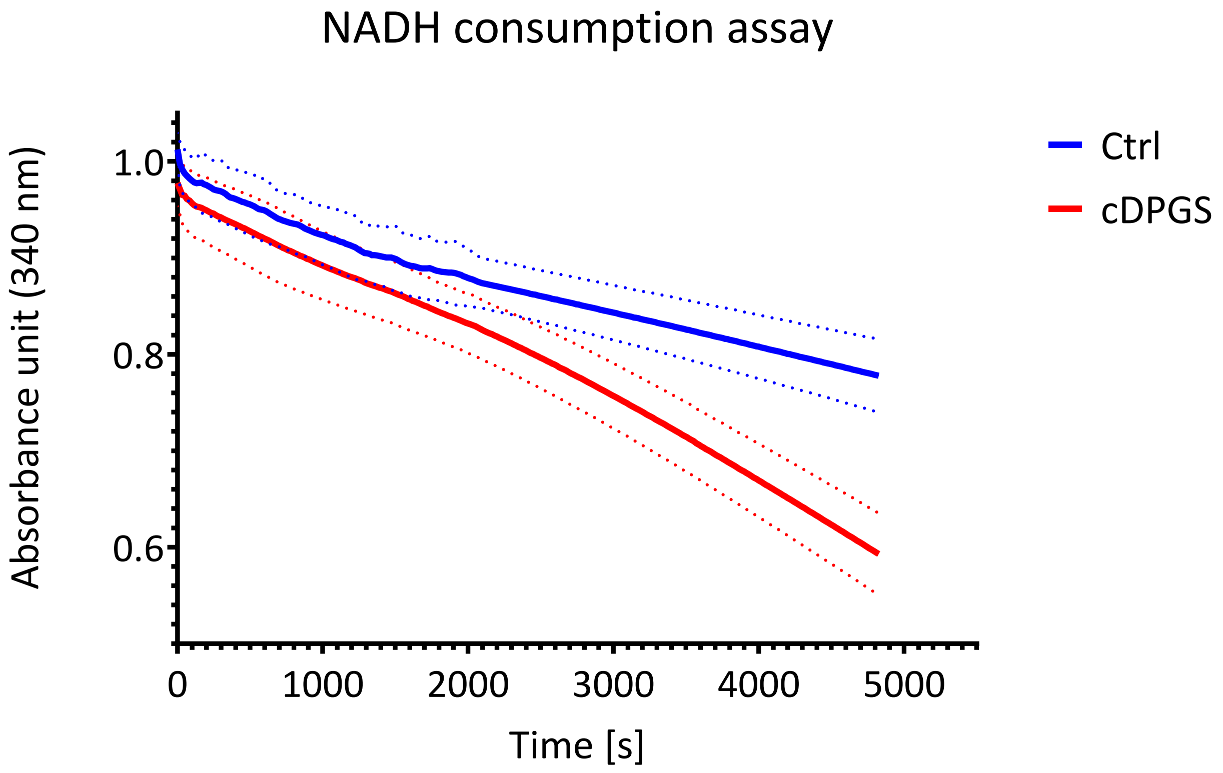


Figure S3: Coupled assay following the consumption of 2,3 DPG. The cDPGS activity was indirectly observed through the amounts of ADP produced from ATP calculated from the respective reactions coupled to the oxidation of NADH using the pyruvate kinase (PK) and lactic acid dehydrogenase (L-LDH) from rabbit muscle. Assays were performed in triplicate and a control reaction was carried out without the enzyme cDPGS, where the total volume was maintained by adding buffer. A background absorbance was observed due to oxidative activity within the assay solution. The error bars are shown as dotted lines.

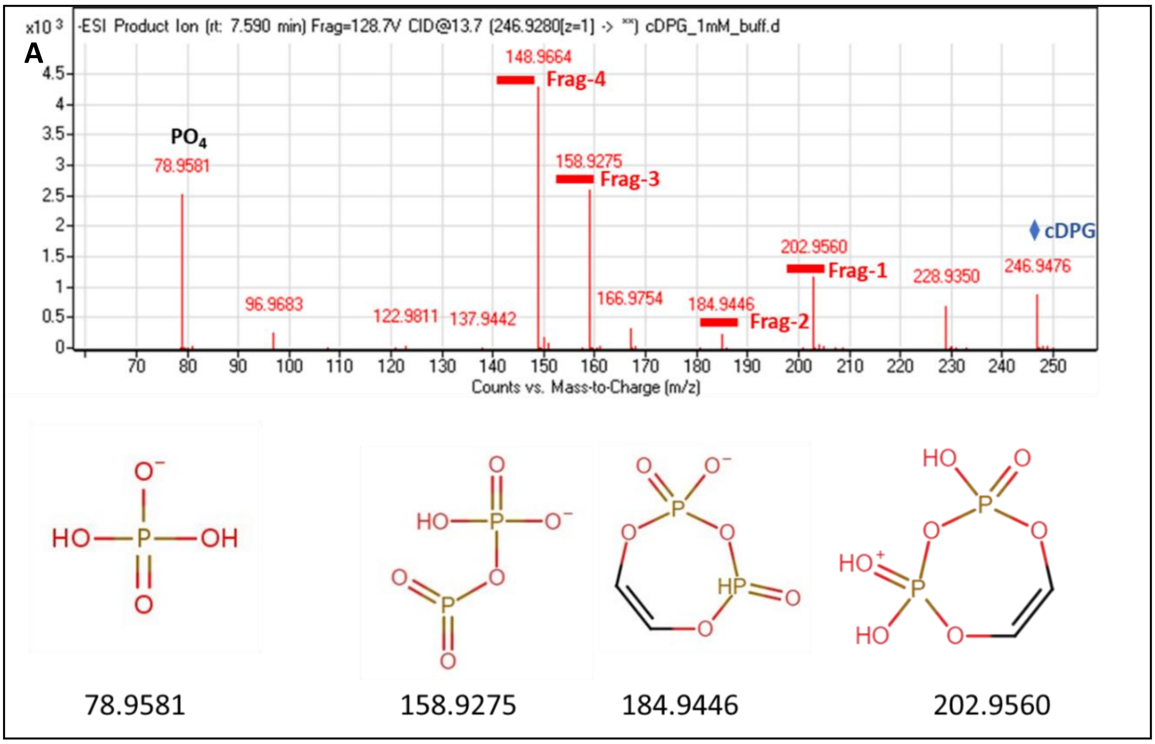


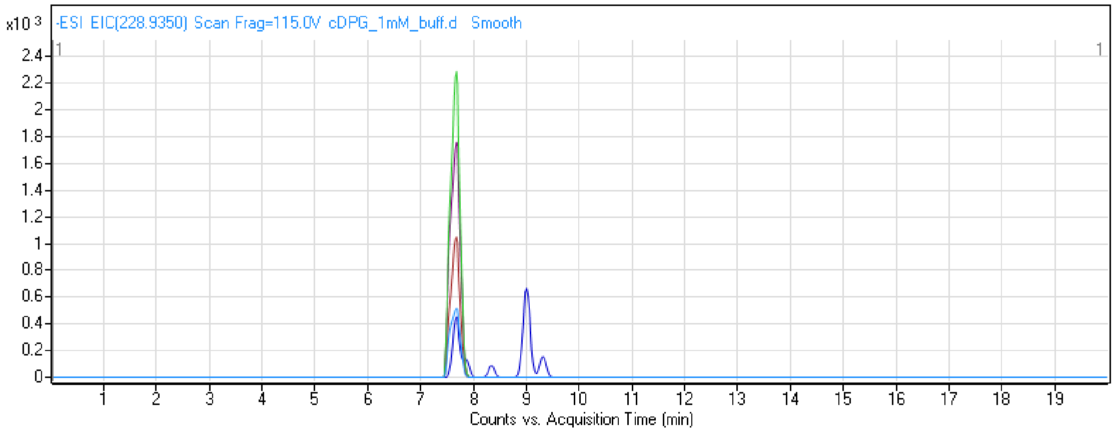


Figure S4: **A)** The MS fragmentation of cDPG, the whole molecule mass (cDPG: 246.9 Da) is indicated by the blue diamond, while the four fragments' masses are underlined in red, the structures of three of the fragments were predicted with CFM-ID and are shown. **B)** Extracted chromatogram of the reaction products at the rt of the cDPG. Green 202.9560, Purple 148.9664, Brown 158.9275, Light Blue 184.9446, Dark blue 78.9581.The structure of the fragment with mass 148.9664 could not be determined.

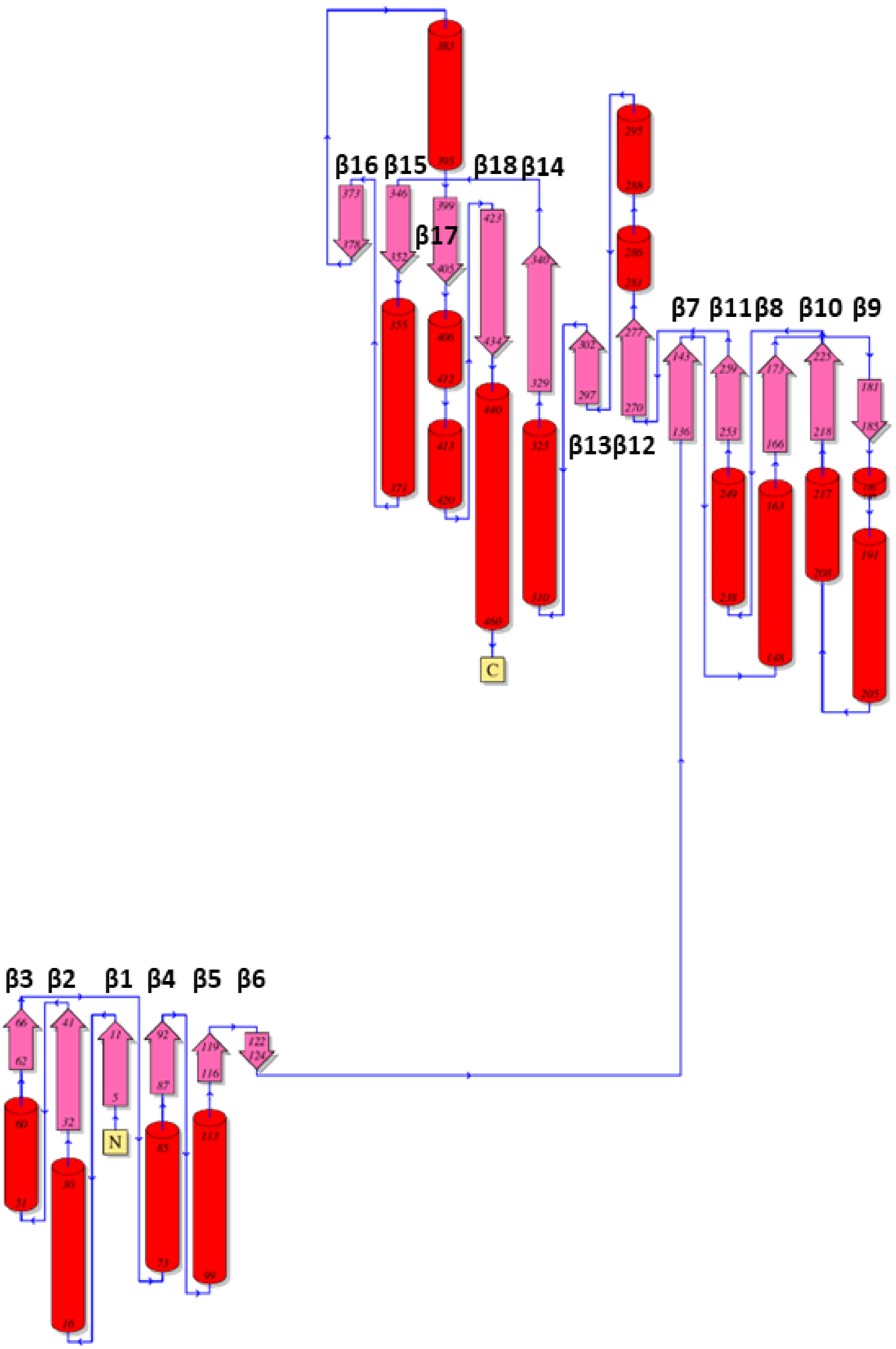


Figure S5: A secondary structure topology diagram of the cDPGS enzyme prepared using LigPlot (Laskowski and Swindells, 2011)

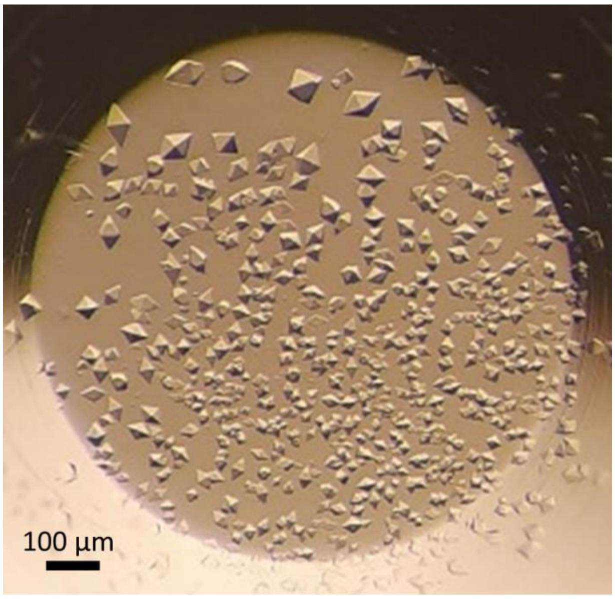


Figure S6: The crystals obtained for the complex with cDPG substrate and ADP bound crystals in condition E12 of the Morpheus™ screen.

Figure S7: Nucleotide sequence of the pLATE51/cDPGS plasmid

```
TAAAACAGGCCTGCATGCAGCGTAATCATGGTCATTAATTAACCTCCTGTGTGAAATTGTTATCCGCTCTGCGTTAGCAA
TTTAACTGTGATAAACTACCGCATTAAAGCTTATCGATGATAAGCTGTCAAACATGAGAAGGATCCTAGCATAACCCCTT
GGGGCCTCTAAACGGGTCTTGAGGGGTTTTTTGCGCCGGCGGACACCATCGAATGGCGCAAAACCTTTCGCGGTATGGCA
TGATAGCGCCCGGAAGAGAGTCAATTCAGGGTGGTGAATGTGAAACCAGTAACGTTATACGATGTCGCAGAGTATGCCGG
TGTCTCTTATCAGACCGTTTCCCGCGTGGTGAACCAGGCCAGCCACGTTTCTGCGAAAACGCGGGAAAAAGTGGAAGCGG
CGATGGCGGAGCTGAATTACATTCCCAACCGCGTGGCACAACAACTGGCGGGCAAACAGTCGTTGCTGATTGGCGTTGCC
ACCTCCAGTCTGGCCCTGCACGCGCCGTCGCAAATTGTCGCGGCGATTAAATCTCGCGCCGATCAACTGGGTGCCAGCGT
GGTGGTGTCGATGGTAGAACGAAGCGGCGTCGAAGCCTGTAAAGCGGCGGTGCACAATCTTCTCGCGCAACGCGTCAGTG
GGCTGATCATTAACTATCCGCTGGATGACCAGGATGCCATTGCTGTGGAAGCTGCCTGCACTAATGTTCCGGCGTTATTT
CTTGATGTCTCTGACCAGACACCCATCAACAGTATTATTTTCTCCCATGAAGACGGTACGCGACTGGGCGTGGAGCATCT
GGTCGCATTGGGTCACCAGCAAATCGCGCTGTTAGCGGGCCCATTAAGTTCTGTCTCGGCGCGTCTGCGTCTGGCTGGCT
GGCATAAATATCTCACTCGCAATCAAATTCAGCCGATAGCGGAACGGGAAGGCGACTGGAGTGCCATGTCCGGTTTTCAA
CAAACCATGCAAATGCTGAATGAGGGCATCGTTCCCACTGCGATGCTGGTTGCCAACGATCAGATGGCGCTGGGCGCAAT
GCGCGCCATTACCGAGTCCGGGCTGCGCGTTGGTGCGGATATCTCGGTAGTGGGATACGACGATACCGAAGACAGCTCAT
GTTATATCCCGCCGTTAACCACCATCAAACAGGATTTTCGCCTGCTGGGGCAAACCAGCGTGGACCGCTTGCTGCAACTC
TCTCAGGGCCAGGCGGTGAAGGGCAATCAGCTGTTGCCCGTCTCACTGGTGAAAAGAAAAACCACCCTGGCGCCCAATAC
GCAAACCGCCTCTCCCCGCGCGTTGGCCGATTCATTAATGCAGCTGGCACGACAGGTTTCCCGACTGGAAAGCGGGCAGT
AATTCCGGATCTGCATCGCAGGATGCTGCTGGCTACCCTGTGGAACACCTACATCTGTATTAACGAAGCGCTGGCATTGA
CCCTGAGTGATTTTTCTCTGGTCCCGCCGCATCCATACCGCCAGTTGTTTACCCTCACAACGTTCCAGTAACCGGGCATG
TTCATCATCAGTAACCCGTATCGTGAGCATCCTCTCTCGTTTCATCGGTATCATTACCCCCATGAACAGAAATCCCCCTT
ACACGGAGGCATCAGTGACCAAACAGGAAAAAACCGCCCTTAACATGGCCCGCTTTATCAGAAGCCAGACATTAACGCTT
CTGGAGAAACTCAACGAGCTGGACGCGGATGAACAGGCAGACATCTGTGAATCGCTTCACGACCACGCTGATGAGCTTTA
CCGCAGCTGCCTCGCGCGTTTCGGTGATGACGGTGAAAACCTCTGACACATGCAGCTCCCGGAGACGGTCACAGCTTGTC
TGTAAGCGGATGCCGGGAGCAGACAAGCCCGTCAGGGCGCGTCAGCGGGTGTTGGCGGGTGTCGGGGCGCAGCCATGACC
CAGTCACGTAGCGATAGCGGAGTGTATACTGGCTTAACTATGCGGCATCAGAGCAGATTGTACTGAGAGTGCACCATATG
CGGTGTGAAATACCGCACAGATGCGTAAGGAGAAAATACCGCATCAGGCGCTCTTCCGCTTCCTCGCTCACTGACTCGCT
GCGCTCGGTCGTTCGGCTGCGGCGAGCGGTATCAGCTCACTCAAAGGCGGTAATACGGTTATCCACAGAATCAGGGGATA
ACGCAGGAAAGAACATGTGAGCAAAAGGCCAGCAAAAGGCCAGGAACCGTAAAAAGGCCGCGTTGCTGGCGTTTTTCCAT
AGGCTCCGCCCCCCTGACGAGCATCACAAAAATCGACGCTCAAGTCAGAGGTGGCGAAACCCGACAGGACTATAAAGATA
CCAGGCGTTTCCCCCTGGAAGCTCCCTCGTGCGCTCTCCTGTTCCGACCCTGCCGCaAAGGATCCGGATACCTGTCCGCC
TTTCTCCCTTCGGGAAGCGTGGCGCTTTCTCATAGCTCACGCTGTAGGTATCTCAGTTCGGTGTAGGTCGTTCGCTCCAA
GCTGGGCTGTGTGCACGAACCCCCCGTTCAGCCCGACCGCTGCGCCTTATCCGGTAACTATCGTCTTGAGTCCAACCCGG
TAAGACACGACTTATCGCCACTGGCAGCAGCCACTGGTAACAGGATTAGCAGAGCGAGGTATGTAGGCGGTGCTACAGAG
```

TTCTTGAAGTGGTGGCCTAACTACGGCTACACTAGAAGGACAGTATTTGGTATCTGCGCTCTGCTGAAGCCAGTTACCTT
CGGAAAAAGAGTTGGTAGCTCTTGATCCGGCAAACAAACCACCGCTGGTAGCGGTGGTTTTTTTGTTTGCAAGCAGCAGA
TTACGCGCAGAAAAAAAGGATCTCAAGAAGATCCTTTGATCTTTTCTACGGGGTCTGACGCTCAGTGGAACGAAAACTCA
CGTTAAGGGATTTTGGTCATGAGATTATCAAAAAGGATCTTCACCTAGATCCTTTTAAATTAAAAATGAAGTTTTAAATC
AATCTAAAGTATATATGAGTAAACTTGGTCTGACAGTTACCAATGCTTAATCAGTGAGGCACCTATCTCAGCGATCTGTC
TATTTCGTTCATCCATAGTTGCCTGACTCCCCGTCGTGTAGATAACTACGATACGGGAGGGCTTACCATCTGGCCCCAGT
GCTGCAATGATACCGCGAGAACCACGCTCACCGGCTCCAGATTTATCAGCAATAAACCAGCCAGCCGGAAGGGCCGAGCG
CAGAAGTGGTCCTGCAACTTTATCCGCCTCCATCCAGTCTATTAATTGTTGCCGGGAAGCTAGAGTAAGTAGTTCGCCAG
TTAATAGTTTGCGCAACGTTGTTGCCATTGCTGCAGGCATCGTGGTGTCACGCTCGTCGTTTGGTATGGCTTCATTCAGC
TCCGGTTCCCAACGATCAAGGCGAGTTACATGATCCCCCATGTTGTGCAAAAAAGCGGTTAGCTCCTTCGGTCCTCCGAT
CGTTGTCAGAAGTAAGTTGGCCGCAGTGTTATCACTCATGGTTATGGCAGCACTGCATAATTCTCTTACTGTCATGCCAT
CCGTAAGATGCTTTTCTGTGACTGGTGAGTACTCAACCAAGTCATTCTGAGAATAGTGTATGCGGCGACCGAGTTGCTCT
TGCCCGGCGTCAACACGGGATAATACCGCGCCACATAGCAGAACTTTAAAAGTGCTCATCATTGGAAAACGTTCTTCGGG
GCGAAAACTCTCAAGGATCTTACCGCTGTTGAGATCCAGTTCGATGTAACCCACTCGTGCACCCAACTGATCTTCAGCAT
CTTTTACTTTCACCAGCGTTTCTGGGTGAGCAAAAACAGGAAGGCAAAATGCCGCAAAAAAGGGAATAAGGGCGACACGG
AAATGTTGAATACTCATACTCTTCCTTTTTCAATATTATTGAAGCATTTATCAGGGTTATTGTCTCATGAGCGGATACAT
ATTTGAATGTATTTAGAAAAATAAACAAATAGGGGTTCCGCGCACATTTCCCCGAAAAGTGCCACCTGACGTCTAAGAAA
CCATTATTATCATGACATTAACCTATAAAAATAGGCGTATCACGAGGCCCTTTGCGCCGGCGAAAACGAAAGGCTCAGTC
GAAAGACTGGGCCTTTCGTTTTATCTGTTGTTTGTCGGTGAACGCTCTCCTGAGTAGGACAAATCCGCCGGGAGCGGATT
TGAACGTTGCGAAGCAACGGCCCGGAGGGTGGCGGGCAGGACGCCCGCCATAAACTGCCAGGCATCAAATTAAGCAGAAG
GCCATCCTGACGGATGGCCTTTTTGAGATCAATTCTTAAATTGTGAGCGGATAACAATTTGAGCTCGGTACCTTTTCGCG
CGCGTTTCCCTGCAGGTGGCGCGCCTGTAATACGACTCACTATAGGGGAATTGTGAGCGGATAACAATTCCCCCTCTAGA
AATAATTTTGTTTAACATTTAAATCCAGAAGGAGATATAACTATGGCGGGTTCTCATCATCATCATCATCATGGTATGGC
TAGCATGACTGGTGGACAGCAAATGGGTCGCTCCCTCGAGATGGGCGAGACAAAAAAGATGATTTGCCTGGTAGATGGGG
AACACTATTTTCCTGTTGTTAAAGACAGCATTGAAATCCTCGATGATCTGGAGCATATCGACGTAGTGGCTGTGGTATTC
ATCGGCGGAACCGAGAAACTGCAGATTGAAGATCCGAAAGAATATTCGGAAAAACTGGGCAAACCTGTGTTCTTTGGACC
CGATCCGAAGAAAATTCCGTATGACGTTATCAAGAAATGCGTCAAGAAATACAATGCGGATATTGTGATGGATCTTTCTG
ACGAACCAGTAGTGGACTACACCAAACGGTTTCGCATCGCCTCCATTGTGCTGAAAGAGGGCGCAGTTTATCAAGGGGCC
GATTTTAAATTTGAACCGCTGACTGAATACGATGTTTTGGAGAAACCGTCTATCAAAATTATTGGTACCGGGAAACGCAT
TGGTAAGACAGCGGTGAGTGCGTATGCAGCCCGTGTGATTCACAAGCATAAATACAATCCCTGTGTAGTTGCAATGGGCC
GTGGTGGACCACGTGAACCGGAGATTGTGGAGGGCAACAAAATCGAAATCACCGCCGAATATCTGCTTGAGCAAGCGGAT
AAAGGCGTTCATGCAGCCAGCGATCATTGGGAAGATGCCCTGATGAGTCGCATTCTGACGGTTGGATGTCGTCGTTGTGG
TGGTGGGATGCTGGGCGACACGTTCATTACCAACGTCAAACGTGGTGCAGAGATTGCGAACAAACTGGACTCAGATTTTG
TCATTATGGAAGGTTCAGGTGCGGCAATTCCGCCGGTGAAAACGAATCGGCAGATTGTCACTGTTGGCGCCAATCAGCCG
ATGATCAACATCAATAACTTCTTTGGCCCGTTTCGCATTGGCTTAGCCGATTTGGTCATCATTACCATGTGTGAAGAACC
GATGGCGACCACCGAAAAGATCAAGAAAGTTGAGAAATTCATTAAAGAGATCAATCCCAGCGCTAATGTGATTCCGACGG
TTTTCCGCCCAAAACCTGTGGGTAACGTCGAAGGTAAAAAAGTGTTGTTTGCGACCACGGCCCCGAAAGTTGTGGTAGGG
AAACTCGTGAATTACCTGGAATCGAAATATGGCTGCGATGTAGTGGGTGTTACGCCACACCTGAGCAATCGCCCTCTGTT
ACGTCGCGATTTAAAGAAATACATTAACAAAGCGGATCTTATGCTCACTGAACTGAAAGCGGCTGCTGTGGATGTCGCGA
CACGCGTAGCTATTGAAGCGGGCTTAGATGTCGTGTATTGCGACAACATCCCAGTCGTCATCGACGAATCCTATGGCAAC
ATTGACGATGCAATCATCGAAGTGGTCGAAATGGCTATCGACGACTTCAAGAACAACCGC

pLATE51-cDPGS.gb

GenBank file of the pLATE51/cDPGS plasmid:
